# A local effect model-based interpolation framework for experimental nanoparticle radiosensitisation data

**DOI:** 10.1186/s12645-016-0025-6

**Published:** 2017-01-31

**Authors:** Jeremy M. C. Brown, Fred J. Currell

**Affiliations:** School of Mathematics and Physics, Queen’s University Belfast, Belfast, Northern Ireland, UK

**Keywords:** Gold nanoparticles, Local effect model (LEM), Radiosensitisers, Radiotherapy, Biological effect modelling

## Abstract

A local effect model (LEM)-based framework capable of interpolating nanoparticle-enhanced photon-irradiated clonogenic cell survival fraction measurements as a function of nanoparticle concentration was developed and experimentally benchmarked for gold nanoparticle (AuNP)-doped bovine aortic endothelial cells (BAECs) under superficial kilovoltage X-ray irradiation. For three different superficial kilovoltage X-ray spectra, the BAEC survival fraction response was predicted for two different AuNP concentrations and compared to experimental data. The ability of the developed framework to predict the cell survival fraction trends is analysed and discussed. This developed framework is intended to fill in the existing gaps of individual cell line response as a function of NP concentration under photon irradiation and assist the scientific community in planning future pre-clinical trials of high Z nanoparticle-enhanced photon radiotherapy.

## Background

Photon radiotherapy has undergone significant evolution with the development of new technologies and increased understanding of radiobiology (Mayles et al. 2007; Joiner and van der Kogel 2009). Over the last 15 years, one of the most promising refinements of this cancer treatment modality has been the development and functionalisation of high Z nanoparticles to target cancerous small animals/humans cell lines (Hainfeld et al. 2004, 2008; Jain et al. 2011). This class of novel nanomedicines, of which gold nanoparticles (AuNP) are the most popular (Jain et al. 2012), is thought to increase the local energy deposition and, in-turn, water radiolysis free-radical yield with a few 10–100 nms surrounding each NP (Jones et al. 2010; McMahon et al. 2011; Lechtman et al. 2013; Lin et al. 2014; Sicard-Roselli et al. 2014; Tran et al. 2016). Whilst this technology is still in development and its exact biological action pathway is under intensive investigation, it has already been shown that NP radiosensitising agents utilised in conjunction with radiotherapy are able to provide increased tumour control and life expectancy in small animal models (Hainfeld et al. 2004, 2013; Joh et al. 2013; Xing et al. 2013).

Development and experimental testing of functionalisation high Z NP radiosensitisers for a given cell line is a complex process which can take significant time and resources. Over the last decade, the scientific community has shifted towards exploring the potential of a developed high Z NP radiosensitiser for photon radiotherapy through mechanistic characterisation utilising radiation transport codes such as EGSnrc (Kawrakow 2000), Geant4/Geant4-DNA (Agostinelli et al. 2003;  Allison et al. 2006, 2016; Incerti et al. 2010; Bernal et al. 2015), MCNPX (Pelowitz 2005) and PENELOPE (Baro et al. 1995; Salvat et al. 2006). Originally, the scientific community tried to predict the increased effect of high Z NPs through the use of a variety of dose enhancement figures of merit (DEFM) known via a number of different names. All of these DEFMs were based on the assumption that expected biological outcome of cells/tumours could be described via the ratio of dose deposition with and without high Z NP doping under uniform photon irradiation (Cho 2005; Roeske et al. 2007; Ngwa et al. 2010). This underlying assumption neglects two of the key physical factors which determine the action of high Z NP within cells under photon irradiation: (1) the increased localised energy deposition within the first few 10–100 nms of the NP surface (Jones et al. 2010; McMahon et al. 2011; Lechtman et al. 2013; Lin et al. 2014; Sicard-Roselli et al. 2014; Tran et al. 2016), and (2) NP distribution within the irradiated cells (Lechtman et al. 2013; Brun et al. 2009; Coulter et al. 2012; Cui et al. 2014; McQuaid et al. 2016). An alternative to these DEFMs, the local effect model (LEM) (Scholz and Kraft 1996, 2004) was first applied 5 years ago to photon radiotherapy in an attempt to account for one of these two key physical factors: the increased dose localisation within the first few 10–100 nm of the NP surface (McMahon et al. 2011). Two years later, Lechtman et al. (2013) proposed an extension specifically for AuNPs, the AuNP radiosensitisation predictive (ARP) model, in an attempt to account for both of these two physical factors neglected via DEFMs (Lechtman et al. 2013). Both these models were shown to be able to predict specific cell survival fraction behaviour under photon irradiation observed through clonogenic assay (McMahon et al. 2011; Lechtman et al. 2013).

The following work builds on the success of the LEM and presents a new experimentally benchmarked framework capable of interpolating NP-enhanced photon-irradiated clonogenic cell survival fraction measurements as a function of NP concentration. This LEM-based framework was developed to fill in the existing gaps of individual cell line response as a function of NP concentration under photon irradiation to assist the scientific community in planning future pre-clinical trials of high Z nanoparticle-enhanced photon radiotherapy.

## Local effect model-based interpolation framework

The developed LEM-based interpolation framework is intended to be used in conjunction with the existing wealth of available experimental survival fraction data for high Z NP-undoped and NP-doped specific cell line studies (Jain et al. 2012). At a minimum each of these studies possesses a set of in vitro clonogenic assays of a cell line undoped and doped with high Z NPs that have been irradiated by a gamma-/X-ray source with a known energy spectra. The following derivation outlines how these data can be interpolated as a function of NP concentration, up to a maximum concentration corresponding to the NP-doped cell line survival data, within the LEM formalism for a given cell line/incident photon energy spectra combination.

The LEM can be constructed utilising three main assumptions. First, the survival fraction of a cellular colony/system under photon irradiation (SF) can be described via a linear-quadratic response:

1 $$\begin{aligned} {\text{SF}}[D] = \exp \left( -\alpha D - \beta D^2\right) \end{aligned}$$

where $\alpha$ and $\beta$ are characteristics of the target cell line, and *D* is the mean dose delivered to the entire volume of the cellular colony/system (McMahon et al. 2011; Douglas and Fowler 1976). Second, that cell “inactivation”, e.g. cell death, can be attributed to the creation of a number of lethal lesions within a sensitive small sub-cellular volume such as the cell nucleus (Scholz and Kraft 1996, 2004). Here, a lethal lesion is defined as the local modification of DNA generated from the direct and indirect action of ionisation radiation (i.e. a double-strand break). And finally, any contribution of sub-lethal damage at distances larger than the order of a few microns is ignored as it is assumed that there is no interaction between distant sites (Scholz and Kraft 1996, 2004).

Using these assumptions, it is possible to describe the survival fraction for a cell under photon irradiation in terms of the mean number of lethal lesions ($\langle N (D) \rangle$):

2 $$\begin{aligned}{\text{SF}}[D] = \exp (-\langle N (D) \rangle ) \end{aligned}$$

and inversely:

3 $$\begin{aligned} \displaystyle \langle N (D) \rangle = -\log ({\text{SF}}[D]). \end{aligned}$$

Within each cell under photon irradiation, lethal lesions are generated inhomogeneously and the probability of their creation is a direct function of local dose deposition. These properties mean that total lesion number in a cell’s sensitive region can be given via integration over its whole volume:

4 $$\begin{aligned} \displaystyle \langle N_{{\text{total}}} (D) \rangle&= \int \frac{-\log ({\text{SF}}[{{d}}(x,y,z)])}{V_{{\text{sens}}}}{\text{d}}V \nonumber \\&= \alpha \int \frac{{ {d}}(x,y,z)}{V_{{\text{sens}}}}{\text{d}}V + \beta \int \frac{{{d}}(x,y,z)^{2}}{V_{{\text{sens}}}}{\text{d}}V \end{aligned}$$

where ${{d}}(x,y,z)$ is the local dose deposited for a given position within the sensitive region of the cell and $V_{{\text{sens}}}$ is the total volume of the sensitive region of interest.

For a cellular colony/system doped with a concentration of high Z NPs (*C*), the LEM framework allows for the total local dose deposition within the sensitive region of the cell to be separated into two parts:

5 $$\begin{aligned} \displaystyle { {d}}(x,y,z) = { {d}}_{\rm U}(x,y,z)+ { {d}}_{{\text{NP}}}(C,x,y,z) \end{aligned}$$

where ${{d}}_{\rm U}(x,y,z)$ and ${{d}}_{{\text{NP}}}(C,x,y,z)$ are the dose distributions generated within the sensitive region from the direct interaction of radiation with the bulk cell and high Z NPs, respectively. With this separation, Eq. 4 can be expressed as:

6 $$\begin{aligned} \displaystyle \langle N_{{\text{total}}} (C,D) \rangle&= \alpha \int \frac{{{d}}_{\rm U}(x,y,z)+{{d}}_{{\text{NP}}}(C,x,y,z)}{V_{{\text{sens}}}}{\text{d}}V \nonumber \\&\quad + \beta \int \frac{\left( {{d}}_{\rm U}(x,y,z)+{{d}}_{{\text{NP}}}(C,x,y,z)\right) ^{2}}{V_{{\text{sens}}}}{\text{d}}V \nonumber \\&= \alpha \int \frac{{{d}}_{\rm U}(x,y,z)}{V_{{\text{sens}}}}{\text{d}}V + \beta \int \frac{{{d}}_{\rm U}(x,y,z)^{2}}{V_{{\text{sens}}}}{\text{d}}V \nonumber \\&\quad + \alpha \int \frac{{{d}}_{{\text{NP}}}(C,x,y,z)}{V_{{\text{sens}}}}{\text{d}}V + \beta \int \frac{{{d}}_{{\text{NP}}}(C,x,y,z)^{2}}{V_{{\text{sens}}}}{\text{d}}V \nonumber \\&\quad + 2\beta \int \frac{{{d}}_{\rm U}(x,y,z)\times {{d}}_{{\text{NP}}}(C,x,y,z)}{V_{{\text{sens}}}}{\text{d}}V. \end{aligned}$$

In addition, over the range of validity of dose in the linear-quadratic model, 1–6 Gy (Joiner and van der Kogel 2009), the probability of two energy deposits within ${{d}}_{\rm U}(x,y,z)$ and ${{d}}_{{\text{NP}}}(C,x,y,z)$ at the same location can be assumed to be negligible. Therefore, their product term in Eq. 6 can be set to zero such that:

7 $$\begin{aligned} \displaystyle \langle N_{{\text{total}}} (C,D) \rangle&\approx \alpha \int \frac{{{d}}_{\rm U}(x,y,z)}{V_{{\text{sens}}}}{\text{d}}V + \beta \int \frac{{{d}}_{\rm U}(x,y,z)^{2}}{V_{{\text{sens}}}}{\text{d}}V \nonumber \\&\quad + \alpha \int \frac{{{d}}_{{\text{NP}}}(C,x,y,z)}{V_{{\text{sens}}}}{\text{d}}V + \beta \int \frac{{{d}}_{{\text{NP}}}(C,x,y,z)^{2}}{V_{{\text{sens}}}}{\text{d}}V \nonumber \\&= \langle N_{U} (D) \rangle + \langle N_{{\text{NP}}} (C,D) \rangle \end{aligned}$$

where $\langle N_{U} (D) \rangle$ is the mean number of lethal lesion generated via photon interaction within an undoped cellular region, and $\langle N_{{\text{NP}}} (C,D) \rangle$ is the mean number of lethal lesion generated via high Z NP action within the doped cellular region. Here, $\langle N_{{\text{NP}}} (C,D) \rangle$ encompasses the lethal lesion generated from direct photon interaction with NPs, secondary electron generated from photon–cellular medium interaction collisions with NPs, and secondary electron/photons generated from photon–NP interactions collision with other NPs. If the spatial distribution of NP uptake within the cell line remains approximately constant with concentration, then from a mechanistic perspective the mean number of lethal lesions generated from these effects can be scaled with average NP density up to a critical saturation threshold (McKinnon et al. 2016). Under these assumptions, Eq. 7 can be manipulated to yield:

8 $$\begin{aligned} \displaystyle \langle N_{{\text{NP}}} (C,D) \rangle&= \langle N_{{\text{total}}} (C,D) \rangle - \langle N_{\rm{U}} (D) \rangle \nonumber \\&\approx \frac{C}{C_{0}}\left( \langle N_{{\text{total}}} (C_{0},D) \rangle - \langle N_{\rm{U}} (D) \rangle \right) \end{aligned}$$

where $\langle N_{{\text{total}}} (C_{0},D) \rangle$ is the mean number of lethal lesions for a given dose *D* at a known reference concentration $C_{0}$. With this, Eq. 7 can be expressed as:

9 $$\begin{aligned} \displaystyle \langle N_{{\text{total}}} (C,D) \rangle&= \langle N_{\rm{U}} (D) \rangle + \frac{C}{C_{0}}\left( \langle N_{{\text{total}}} (C_{0},D) \rangle - \langle N_{\rm{U}} (D) \rangle \right) \nonumber \\&= -\log ({\text{SF}}_{\rm{U}}[D]) - \frac{C}{C_{0}}\left( \log ({\text{SF}}_{{\text{total}}}[C_{0},D])-\log ({\text{SF}}_{\rm{U}}[D])\right) \nonumber \\&= \left( \alpha _{\rm{U}}+\frac{C}{C_{0}}\Delta \alpha \right) D + \left( \beta _{\rm{U}}+\frac{C}{C_{0}}\Delta \beta \right) D^{2} \end{aligned}$$

where $\Delta \alpha = \alpha _{{\text{total}}}(C_{0}) - \alpha _{\rm{U}}$ and $\Delta \beta = \beta _{{\text{total}}}(C_{0}) - \beta _{\rm{U}}$. The final form of the interpolation framework is then given via the substitution of Eq. 9 into Eq. 2:

10 $$\begin{aligned} \displaystyle {\text{SF}}[C,D] = \exp \left( -\left( \alpha _{\rm{U}}+\frac{C}{C_{0}}\Delta \alpha \right) D - \left( \beta _{\rm{U}}+\frac{C}{C_{0}}\Delta \beta \right) D^{2} \right) . \end{aligned}$$

## Multiple concentration and incident photon spectra experimental benchmarking

**Fig. 1:**
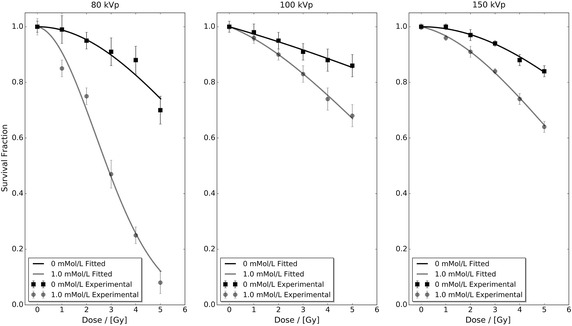
Bovine aortic endothelial cell (BAEC) cell survival fraction as a function of administered 1.9 nm AuNP concentration (0 and 1.0 mMol/L), dose and incident photon spectra (80, 100 and 150 kVp) obtained using a superficial X-ray therapy (SXRT) machine (Therapax 3 Series, Pantak Inc., Branford, CT, USA) at the William Buckland Radiotherapy Centre (The Alfred Hospital, Australia) (Rahman 2010). Data were sourced from the Ph.D. thesis of Rahman (2010)

Experimental benchmarking of the develop framework was undertaken using the only published multiple concentration and incident photon spectra experimental NP radiosensitisation study; the Ph.D. thesis of Rahman, RMIT University (Australia) (Rahman 2010). Within this thesis the radiosensitisation of 1.9 nm AuNP (Nanoprobes Inc., Yaphank, NY 11980, USA) in Bovine Aortic Endothelial Cells (BAECs) under superficial kilovoltage X-ray was studied as a surrogate model for human endothelial cells. The radiosensitivity of four different AuNP concentrations (0, 0.25, 0.5 and 1.0 mMol/L) was explored in triplicate trials for three different kilovoltage X-ray spectra (80, 100 and 150 kVp) delivered via a superficial X-ray therapy (SXRT) machine (Therapax 3 Series, Pantak Inc., Branford, CT, USA) at the William Buckland Radiotherapy Centre (The Alfred Hospital, Australiaρ) (Rahman 2010). Each of these 12 different cell survival curves were composed of a control and five different dose irradiations that were assessed via a CellTiter 96 AQueous One Solution Cell Proliferation Assay (Promega Corp., Madison, Wisconsin). The mean survival fraction, uncertainty (± cell survival standard deviation) and fitted linear-quadratic response of the control (0 mMol/L) and highest concentration (1 mMol/L) data for all three different incident photon spectra are presented in Fig. 1. Each data set’s linear-quadratic response was fitted using least-squares regression in Python, restricting $\alpha$ and $\beta$ to positive values, and their corresponding parameters can be found in Table 1. Further information regarding experimental procedure, AuNP cellular localisation, AuNP cytotoxicity, cell viability, and cell mobility can be found in Rahman’s thesis (Rahman 2010).

**Table 1:** Linear-quadratic parameters for each cell survival curve shown in Fig. 1

Photon spectra (kVp)	Concentration (mMol/L)	$\alpha$ (Gy$^{-1}$)	$\beta$ (Gy$^{-2}$)
80	0.00	0.00 ± 1.66 × 10^−2 ^	1.96 × 10^−2^ ± 4.36 × 10^−3^
	1.00	1.58 × 10^−2^ ± 4.64 × 10^−2^	8.10 × 10^−2^ ± 1.67 × 10^−2^
100	0.00	2.52 × 10^−2^ ± 3.69 × 10^−3^	1.30 × 10^−3^ ± 9.39 × 10^−4^
	1.00	3.47 × 10^−2^ ± 3.36 × 10^−3^	9.04 × 10^−3^ ± 9.64 × 10^−4^
150	0.00	0.00 ± 3.95 × 10^−3^	7.14 × 10^−3^ ± 1.08 × 10^−3^
	1.00	2.03 × 10^−2^ ± 4.44 × 10^−3^	1.34 × 10^−2^ ± 1.26 × 10^−3^

Each data set was fitted using least-squares regression in Python whilst restricting $\alpha$ and $\beta$ to positive values

**Fig. 2:**
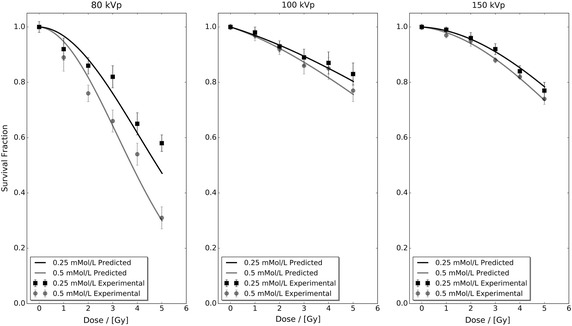
Predicted and extracted experimental bovine aortic endothelial cell (BAEC) survival fractions for 0.25 and 0.5 mMol/L administered 1.9 nm AuNP under 80, 100 and 150 kVp superficial X-ray irradiation. The predicted data sets were calculated utilising Eq. 10 and cell survival fitted linear-quadratic parameters presented in Table 1

The developed interpolation framework was applied to the control and AuNP-doped fitted linear-quadratic parameters contained in Table 1 to predict the BAEC survival fraction response as a function of dose for AuNP concentrations of 0.25 and 0.5 mMol/L for all three different incident photon spectra. Figure 2 presents these predicted data sets in conjunction with the 0.25 and 0.5 mMol/L experimental data from Rahman (2010). Comparison of the predicted response and experimental data sets shows that the developed interpolation framework is able to accurately predict the BAEC survival fraction response to within experimental uncertainties for all dose points in the 100 and 150 kVp data sets. For the 80 kVp data, the predicted survival fraction response is within experimental uncertainty for three data points out of six in both the tested 0.25 and 0.5 mMol/L cases. This poor performance of the developed interpolation framework at 80 kVp can be attributed to the high level of statistical fluctuation within the base 80 kVp experimental data seen in Fig. 1.

**Fig. 3:**
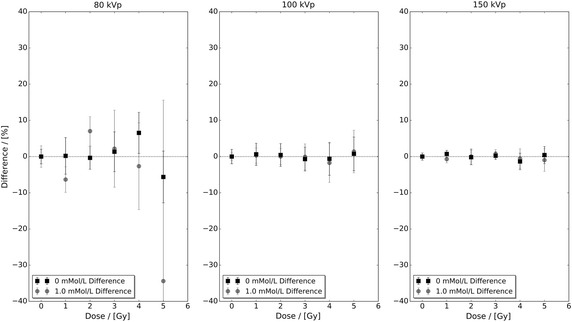
The percentage difference between the control and highest concentration experimental data sets with respect to their fitted linear-quadratic responses shown in Fig. 1. The observed level of difference in the 80 kVp data exceeds both the 100 and 150 kVp data

**Fig. 4:**
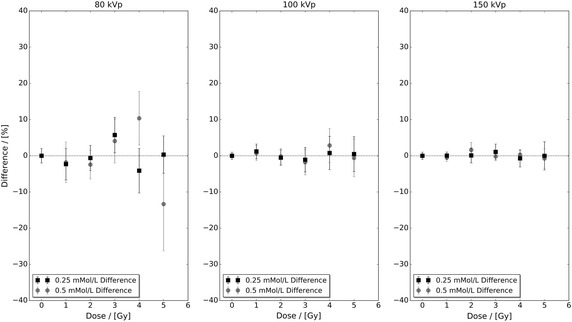
The percentage difference of the 0.25 and 0.5 mMol/L experimental data in Fig. 2 with respect to their fitted linear-quadratic responses obtained utilising the same protocols as Table 1. The level of difference in the 80 kVp data exceeds both the 100 and 150 kVp data as it did for the control and highest concentration experimental data sets seen in Fig. 3

Figure 3 presents the percentage difference between the control and highest concentration experimental data sets with respect to their fitted linear-quadratic responses shown in Fig. 1. In this figure, it can be seen that the level of difference in the 80 kVp data exceeds both the 100 and 150 kVp data sets. However, the magnitude of the observed difference in Fig. 2 cannot be explained via Fig. 3 alone. Figure 4 presents the percentage difference of the 0.25 and 0.5 mMol/L experimental data in Fig. 2 with respect to their fitted linear-quadratic responses obtained utilising the same protocols as Table 1. The level of difference in the 80 kVp data again exceeds the 100 and 150 kVp data sets, and their combined respective magnitudes with those seen in Fig. 3 correlate with the observation deviation between the experimental and predicted 80 kVp data seen in Fig. 2. These observations indicate that the performance of the developed interpolation framework is directly dependent on the quality of input data, a characteristic common to many interpolative frameworks.

## Discussion

A LEM-based framework capable of interpolating NP-enhanced photon irradiated clonogenic cell survival fraction measurements as a function of NP concentration was developed and experimentally benchmarked for 1.9 nm AuNP-doped BAECs under superficial kilovoltage X-ray irradiation. It was illustrated that the performance of the developed framework is directly dependent on the quality of input experimental data. However, further inspection of the percentage differences between experimental data and their respective fitted linear-quadratic responses shown in Figs. 3 and 4 also illustrates that there are limits to which statistical fluctuation can be suppressed via a linear-quadratic fitting approach. Another observation with respect to linear-quadratic response fitting and the present work is that the resultant $\alpha$ and $\beta$ values must be restricted to being positive. Without these restrictions, the predicted survival fraction response would be incorrectly estimated. For example, if either value of $\alpha _{\text{total}}(C_{0})$ or $\beta _{\text{total}}(C_{0})$ was negative, it would result in an underestimation of the predicted survival fraction response. Whereas if either value of $\alpha _{\rm{U}}$ or $\beta _{\rm{U}}$ was negative, it would result in an overestimation of the predicted survival fraction response. Either of these outcomes in the context of high Z NP-enhanced photon radiotherapy treatment planning is unacceptable as it would pose a significant risk to the patient.

The LEM-based interpolation framework presented in this work was developed to fill in the existing gaps within individual cell line response data as a function of NP concentration under photon irradiation. These interpolated data sets will be used in conjunction with another predictive framework that has been developed at Queen’s University Belfast which expresses the enhanced biological response of NP-doped cells/systems in terms of standard photon radiotherapy dose. These two predictive frameworks form the basis of a novel methodology which is intended to assist the scientific community in planning future pre-clinical trials of high Z NP-enhanced photon radiotherapy. Further work is presently underway to illustrate the potential of these two frameworks in the context of AuNP-enhanced breast cancer MV photon radiotherapy as a medical exemplar.

## Conclusion

A LEM-based framework capable of interpolating NP-enhanced photon irradiated clonogenic cell survival fraction measurements as a function of NP concentration was developed and experimentally benchmarked for 1.9 nm AuNP-doped BAECs under superficial kilovoltage X-ray irradiation. For three different superficial kilovoltage X-ray spectra (80, 100 and 150 kVp), the BAEC survival fraction response was predicted for two different AuNP concentrations (0.25 and 0.5 mMol/L). Two of the three predicted spectra data sets (100 and 150 kVp) were within experimental uncertainties for all data points, whereas the other data set (80 kVp) was within experimental uncertainties half of the time. The observed poor performance for the 80 kVp data set was found to be due to a high level of statistical fluctuation within the base data and this illustrated that the performance of developed interpolation framework is directly dependent on the quality of the input experimental data. It is anticipated that this interpolation framework will serve as an important tool for planning future pre-clinical and clinical trials of high Z NP-enhanced photon radiotherapy.

## Abbreviations

ARP Model: gold nanoparticle radiosensitisation predictive model
AuNP: gold nanoparticle
BAEC: bovine aortic endothelial cell
DEFM: dose enhancement figures of merit
DNA: deoxyribonucleic acid
LEM: local effect model
NP: nanoparticle
